# DeepSeedling: deep convolutional network and Kalman filter for plant seedling detection and counting in the field

**DOI:** 10.1186/s13007-019-0528-3

**Published:** 2019-11-23

**Authors:** Yu Jiang, Changying Li, Andrew H. Paterson, Jon S. Robertson

**Affiliations:** 1 School of Electrical and Computer Engineering, College of Engineering, The University of Georgia, Athens, 30602 USA; 2 Franklin College of Arts and Sciences, The University of Georgia, Athens, 30602 USA; 3 College of Agricultural & Environmental Sciences, The University of Georgia, Athens, 30602 USA

**Keywords:** Faster RCNN, Object detection, Video tracking, Cotton, Population density

## Abstract

**Background:**

Plant population density is an important factor for agricultural production systems due to its substantial influence on crop yield and quality. Traditionally, plant population density is estimated by using either field assessment or a germination-test-based approach. These approaches can be laborious and inaccurate. Recent advances in deep learning provide new tools to solve challenging computer vision tasks such as object detection, which can be used for detecting and counting plant seedlings in the field. The goal of this study was to develop a deep-learning-based approach to count plant seedlings in the field.

**Results:**

Overall, the final detection model achieved F1 scores of 0.727 (at $IOU_{all}$) and 0.969 (at $IOU_{0.5}$) on the $Seedling_{All}$ testing set in which images had large variations, indicating the efficacy of the Faster RCNN model with the Inception ResNet v2 feature extractor for seedling detection. Ablation experiments showed that training data complexity substantially affected model generalizability, transfer learning efficiency, and detection performance improvements due to increased training sample size. Generally, the seedling counts by the developed method were highly correlated ($R^2$ = 0.98) with that found through human field assessment for 75 test videos collected in multiple locations during multiple years, indicating the accuracy of the developed approach. Further experiments showed that the counting accuracy was largely affected by the detection accuracy: the developed approach provided good counting performance for unknown datasets as long as detection models were well generalized to those datasets.

**Conclusion:**

The developed deep-learning-based approach can accurately count plant seedlings in the field. Seedling detection models trained in this study and the annotated images can be used by the research community and the cotton industry to further the development of solutions for seedling detection and counting.

## Introduction

Plant population density is defined as the number of plant stands per unit area, which is an important factor for agricultural production systems due to its substantial influence on crop yield potential and fruit quality [1–5]. Plant population density is particularly important for growers right after the germination stage, providing hard date from which to evaluate the necessity of replanting the field if the density is not adequate. Thus, it is crucial to calculate the plant population density when plants are in the seedling stage.

To estimate plant population density, a field assessment involves manually counting the number of plant seedlings/stands in randomly selected subareas of a field and using the average value to represent the plant population density. Quadrat and plot-less sampling methods are typically used for subarea sampling [6]. The quadrat sampling uses a quadrat delimiting an area in which seedlings/plant stands can be counted, whereas the plot-less sampling defines a segment with a known length for seedling/stand counting. Both sampling methods require proper configuration (e.g., quadrat size, segment length, and the number of sampling replications) for an optimal estimation accuracy. While subsampling-based approaches are straightforward, they are laborious (especially for large fields) and could be inaccurate if subsampling is not appropriate.

To address these issues, several studies have been conducted to investigate the use of color images to count plant seedlings in the field [7–9]. These studies relied on conventional image processing, which primarily utilized color information to segment vegetative areas that were used for estimation of plant counts. While these approaches showed high counting accuracies (approximately 90%), they had two major disadvantages. First, color information was sensitive to ambient illumination and plant status. For instance, plants looked darker on cloudy days than sunny days, and plants just sprouting from the soil might have different color than well-established seedlings. Second, counting models were site- and time-dependent. Typically, a calibration step was necessary: a small portion of a field would be manually counted to establish a regression model between pixel counts (or the number of segmented areas) and actual plant counts, so the regression model could be applied to the rest of the images for automatic processing. A regression model established in one experimental site (growth stage) might not transfer to another site (growth stage), requiring model validation or re-calibration. In particular, breeding programs and genetics studies involve a wide variety of genotypes with considerable variations in germination time, raising a particular concern about using these image-processing-based approaches for plant counting.

Seedling detection is an essential part of seedling counting. Recent breakthroughs in deep learning (e.g., deep convolutional neural networks, also known as, CNNs) have demonstrated strong performance for object detection [10]. In particular, faster-region-based CNN (Faster-RCNN) was developed as a CNN-based meta-architecture for object detection [11], which has been shown to provide state-of-the-art performance for various applications and competitions [10]. Researchers explored the use of Faster RCNN for in vivo fruit detection for peppers [12, 13], mangoes [14], apples [15, 16], almonds [17], and maize ears [18]. These studies reported promising detection accuracy (F1 score from 0.8 to 0.92) and thus per-image counting accuracy (relative counting errors from 2 to 15%). In addition, several of these studies further expanded the Faster-RCNN-based fruit detection and counting from a single image to consecutive image sequences.

The key challenge of counting in image sequences or videos is to preclude repeated counting of one fruit object. Three approaches were used to solve this challenge. The first approach reconstructed 3D point clouds of a crop row using 2D images by the structure from motion (SfM) technique, and fruit detections were projected from individual 2D images to the reconstructed global 3D space [15, 16]. As a single fruit object occupied a unique 3D position, detections of one fruit object in different 2D images would highly overlap in the 3D space. Thus, redundant detections of one fruit object could be removed to avoid repeated counting. The second approach used the position (from RTK GPS) and pose (from IMU devices) of image acquisition to estimate the geometric correspondence between pixels in two consecutive images. With this approach, fruit detections in one image could be associated with detections in the next image, thus tracking individual detections through image sequences or videos for counting [14]. In the third approach, a tracking-via-detection strategy was developed to track and count fruit objects in image sequences [13, 16]. The key of the tracking-via-detection strategy is detection-tracker association (assign a detection to a tracker). In [13], the intersection over union (IOU) and boundary measure (the ratio of the intersection between a tracker and a detection to the area of that detection) metrics were used to quantify the closeness between a detection and a tracker. Thresholds of IOU and boundary measure were determined using a small set of image sequences. For a given pair of detection and tracker, if they had an IOU value and a boundary measure that exceeded the predetermined thresholds, the detection and tracker would be associated. In [16], optical flow was calculated to estimate object motion (center positions for trackers) between consecutive images. The estimated center positions for trackers would be compared with center positions for detections. If the center-to-center distance was the minimum, a detection and a tracker would be associated. Although all the three approaches provided fairly high counting accuracies (95.56% to 97.83% for [15], 98% for [14], and 95.9% for [13]), they had various limitations. The first approach was computationally expensive due to the SfM-based reconstruction. In addition, certain environmental factors (e.g., wind) would result in failure of 3D reconstruction using the SfM. The second approach was less computationally expensive than the first one, but the use of positioning sensors (e.g., RTK GPS and IMU) substantially increased the cost of data acquisition systems, which could be problematic for small research projects/farms. The third approach was the least expensive in terms of computation and hardware investment, but the tracking strategy was not robust to different noises. For the method used in [13], the IOU and boundary measure thresholds were determined using only a small set of image sequences. If the testing image sequences and videos were acquired in slightly different conditions, the thresholds might become invalid and result in degraded performance. For the optical flow method [16], the calculation of optical flow could be dramatically influenced by ambient illumination changes, resulting in inaccurate motion estimation and tracking [19]. These issues could be addressed by using other tracking methods such as Kalman filter.

To the best of our knowledge, no study has reported the use of a CNN-based approach for seedling detection and counting. Based on the successes of fruit detection and counting, it is worth exploring the use of CNN-based approaches for seedling detection and counting. In particular, the combination of CNN-based detection models and sophisticated tracking framework would provide inexpensive but accurate counting solutions.

The overall goal of this study was to develop an approach based on CNN and Kalman filter to counting cotton seedlings in the field. Specific objectives were to (1) collect and annotate image datasets for detection model training and testing, (2) train Faster-RCNN models for seedling detection, (3) examine the key factors (training sample size, transfer learning efficiency, and generalizability) for detection model training, and (4) use the trained Faster-RCNN models and Kalman filter to track and count cotton seedlings in videos of individual plots or field segments.

## Results

### Detection performance on $Seedling_{All}$ dataset

**Fig. 1:**
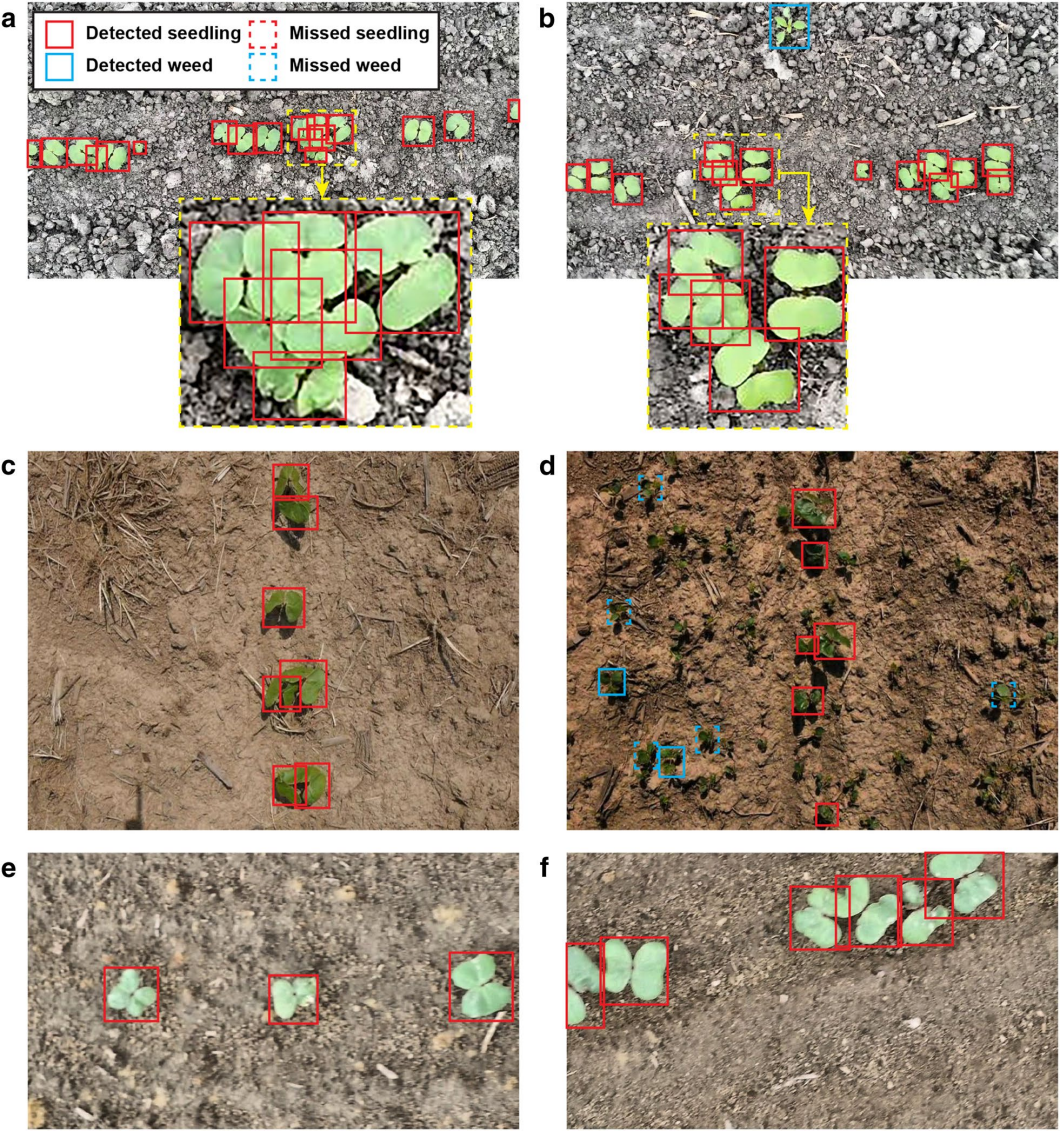
Cotton plant seedlings and weeds detected in representative images of the $Seedling_{All}$ testing set by the Faster RCNN model that was trained using the $Seedling_{All}$ training set. **a**, **b** Are images originally collected in the TAMU2015 dataset, presenting challenges of high seedling occlusion; **c**, **d** in the UGA2015 dataset, presenting challenges of extreme illumination; and **e**, **f** in the UGA2018 dataset without critical challenges for object detection

Overall, the Faster RCNN model ($model_{SAll}$) trained using the $Seedling_{All}$ training set achieved an F1 score ($\frac{2\times precision \times recall}{precision + recall}$) of 0.727 (at $IOU_{all}$) and 0.969 (at $IOU_{0.5}$) on the $Seedling_{All}$ testing set in which images had large variations. The $model_{SAll}$ had even a better performance (F1 score of 0.998) for the seedling class, indicating the efficacy of the Faster RCNN model with an Inception ResNet v2 feature extractor for seedling detection. The $model_{SAll}$ successfully addressed various challenges in the testing images (Fig. 1). The primary challenge in the testing images originally collected in the TAMU2015 dataset was occlusion. Despite excessive overlap between seedling objects, the $model_{SAll}$ could detect all seedlings with corresponding bounding boxes that were tightly fitted to the seedlings (Fig. 1a, b). The key challenges in testing the originally collected images in the UGA2015 dataset were the background complexity and presence of dicotyledonous weeds. The background was relatively simple (no weeds) in some images but could be complex (many weeds) in other images (compare Fig. 1c, d). Seedings were accurately detected by the $model_{SAll}$ even under shaded conditions (the second topmost detection in Fig. 1d). However, weeds (especially small-sized weeds) were not correctly detected when the background was very complex (dashed rectangles in Fig. 1d). It should be noted that there was no misclassification between dicotyledonous weeds and cotton seedlings, both of which are similar in appearance (e.g., color and shape). The RPN network, however, was probably insufficient to provide proposals for regions of interest (ROIs) of small-sized weeds. Nonetheless, the trained models would be acceptable as the primary goal of this study was to detect seedlings rather than weeds.

### Ablation experiment results

#### Training sample size

**Fig. 2:**
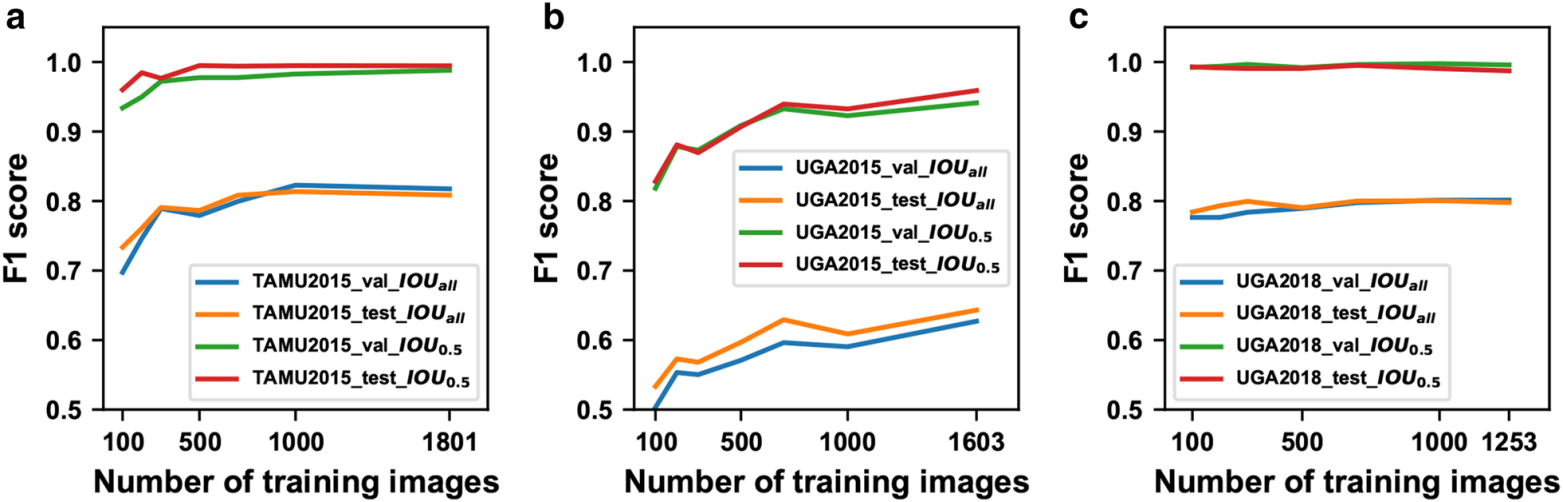
Detection performance (F1 score) calculated using different number of training images for: **a** the TAMU2015 dataset, **b** the UGA2015 dataset, and **c** the UGA2018 dataset. When $\text {IOU}_{\text {all}}$ (a more strict metric) was used, increasing trends of model performance were clearly observed by using more training images

**Fig. 3:**
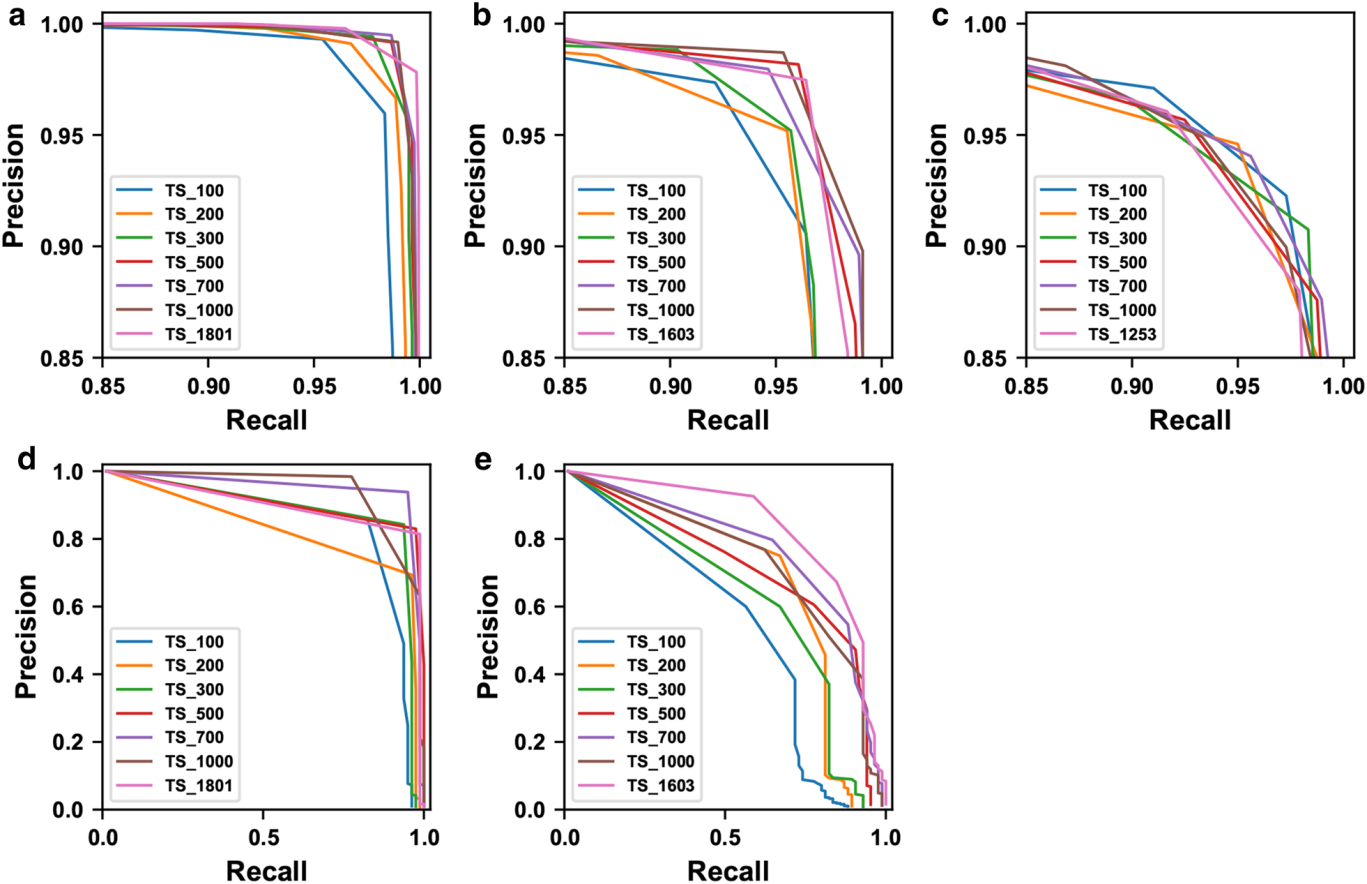
Per-category precision-recall curves generated using different number of training images. **a**–**c** Are for seedling detection in the TAMU2015, UGA2015, and UGA2018 datasets, and **d**, **e** are for weed detection in the TAMU2015 and UGA2015 datasets. A perfect test would have a PR curve that passes through the upper right corner (i.e., 100% for both precision and recall)

Generally, model performance was improved with the increasing of training sample size, but the improvements heavily depended on the evaluation metric and task difficulty (Fig. 2). $IOU_{all}$ is more strict than $IOU_{0.5}$, forcing a higher model accuracy for object localization. Compared with $IOU_{0.5}$, it was clearer to observe performance improvements due to increased training sample size at $IOU_{all}$. The model ($model_{TAMU2015}$) trained on the TAMU2015 dataset reached the maximum F1 score after 500 and 1000 training images at $IOU_{0.5}$ and $IOU_{all}$, respectively (Fig. 2a). The model ($model_{UGA2018}$) trained on the UGA2018 dataset showed a slightly increasing trend at $IOU_{all}$ (compare the curves at $IOU_{0.5}$ and $IOU_{all}$ in Fig. 2c). In addition, the difficulties of seedling and weed detection were different in various images. Based on the results of the $Seedling_{All}$ dataset, seedling and weed detections were the most difficult in the images from the UGA2015 dataset, followed by the TAMU2015 and UGA2018 datasets. When using the same evaluation metric, the increasing trend of model performance with higher number of images was evident for the UGA2015 dataset but less obvious for the TAMU2015 and UGA2018 datasets. This finding held true for individual classes (Fig. 3). For the seedling class, datasets containing challenging situations (e.g., excessive occlusion in the TAMU2015 dataset and high similarities between classes in the UGA2015 dataset) required more than 300 training images to reach the best performance, whereas less-challenging datasets could use only 100 training images for the best result. For the weed class, the precision-recall (PR) curves mostly expanded to be closer to the ideal PR curve (the top-right border) when increasing the training sample size. It was also noteworthy that the expansion magnitudes were more substantial for the UGA2015 dataset than the TAMU2015 dataset.

#### Transfer learning using different pretrained models

**Fig. 4:**
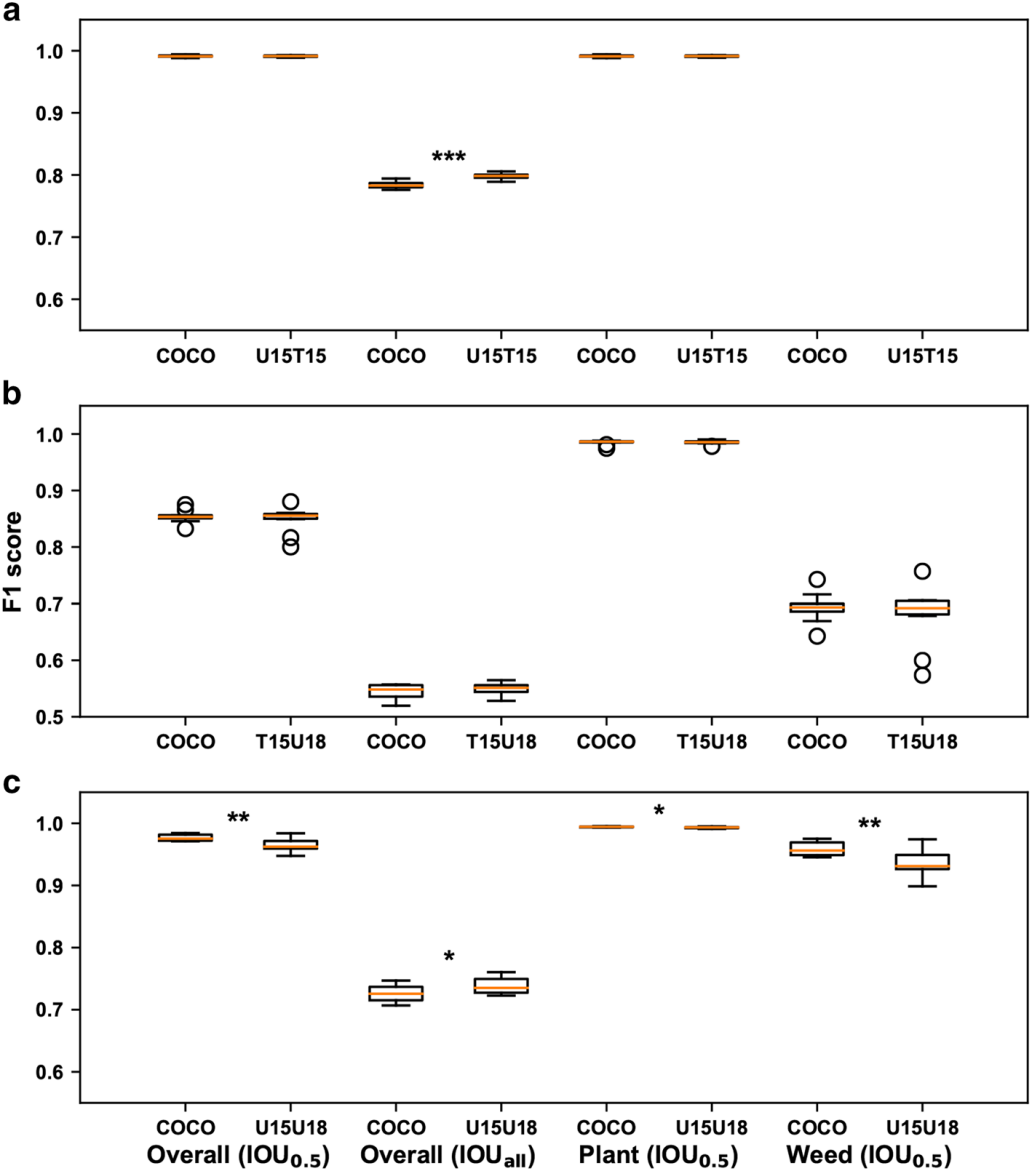
Boxplots of performance (F1 score) on the testing set for models initialized using different pretrained models. **a** Are results for the UGA2018 dataset using models initialized by weights pretrained on the COCO and T15U15 datasets, respectively, **b** are results for the UGA2015 dataset using models initialized by weights pretrained on the COCO and T15U18 datasets, respectively, and **c** are results for the TAMU2015 dataset using models initialized by weights pretrained on the COCO and U15U18 datasets, respectively. Base indicates model initialization using weights pretrained on the COCO dataset, whereas DA indicates model initialization using weights pretrained on a domain dataset. For each of the TAMU2015, UGA2015, and UGA2018 datasets, a subset of 100 images were randomly selected from the training set to train a Faster RCNN model. A total of 10 models were obtained through 10 training repetitions for statistical comparisons between the models. Asterisks indicate statistical differences in model performance at the significance levels of 0.05 (*), 0.01 (**), and less than 0.001 (***)

Transfer learning by model initialization using weights pretrained on a domain dataset showed varied efficiencies. These efficiency variations were dependent on evaluation metrics and datasets (Fig. 4). When using a strict evaluation metric, model initialization using weights pretrained on a domain dataset generally yielded better performance than that using weights pretrained on a common dataset such as the common objects in context (COCO) dataset (overall performance at $IOU_{all}$ in Fig. 4). An exception was identified for the experiment on the TAMU2015 dataset: the overall F1 score from the model initialized using weights pretrained on the COCO dataset was slightly higher than that on the U15U18 dataset (compare the overall performance at $IOU_{0.5}$ and $IOU_{all}$ in Fig. 4c).

#### Model generalizability

The generalizability of trained Faster RCNN models was largely data dependent. The Faster RCNN model trained using the T15U15 dataset ($model_{T15U15}$) provided comparable performance (1% difference) to the $model_{UGA2018}$ for the UGA2018 testing set, indicating a strong model generalizability to new datasets (see results for the UGA2018 testing set in Table 1). On the contrary, Faster RCNN models trained using the U15U18 ($model_{U15U18}$) and T15U18 ($model_{T15U18}$) datasets showed a substantially decreased performance for the testing set of TAMU2015 (F1 score reduction of 36% and 30% at $\text {IOU}_{\text {all}}$ and $\text {IOU}_{\text {0.5}}$) and UGA2015 (F1 score reduction of 41% and 49% at $\text {IOU}_{\text {all}}$ and $\text {IOU}_{\text {0.5}}$) respectively. Although both the two models showed a performance decline for weed detection, the $model_{U15U18}$ showed an acceptable generalizability (F1 score reduction of 7%) for seedling detection, whereas the $model_{T15U18}$ had poor performance of seedling detection (F1 score reduction of 26%) as well (Table 2).

**Table 1:** Overall performance of the model generalizability

Training set	Testing set	mAP $\text {IOU}_{\text {all}}$	mAR100 $\text {IOU}_{\text {all}}$	F1 $\text {IOU}_{\text {all}}$	mAP $\text {IOU}_{\text {0.5}}$	mAR100 $\text {IOU}_{\text {0.5}}$	F1 $\text {IOU}_{\text {0.5}}$
T15U15 training	UGA2018 testing	0.763	0.808	0.785	0.981	1.000	0.991
UGA2018 training	UGA2018 testing	0.778	0.818	0.798	0.983	0.992	0.987
T15U18 training	UGA2015 testing	0.179	0.335	0.233	0.352	0.683	0.464
UGA2015 training	UGA2015 testing	0.599	0.695	0.643	0.923	0.997	0.959
U15U18 training	TAMU2015 testing	0.377	0.535	0.442	0.588	0.857	0.698
TAMU2015 training	TAMU2015 testing	0.791	0.827	0.809	0.989	1.000	0.995

For the testing set in each of the UGA2018, UGA2015, and TAMU2015 datasets, two Faster RCNN models were trained using the training set from the same dataset and from the combination of the other two datasets, respectively. Performance comparison of the two models was used to evaluate the model generalizability

**Table 2:** Per-category performance of the model generalizability at the $\text {IOU}_{\text {0.5}}$

Training set	Testing set	Plant AP	Plant AR100	Plant F1	Weed AP	Weed AR100	Weed F1
T15U15 training	UGA2018 testing	0.981	1.000	0.991	NA	NA	NA
UGA2018 training	UGA2018 testing	0.983	0.992	0.987	NA	NA	NA
T15U18 training	UGA2015 testing	0.676	0.814	0.739	0.027	0.553	0.052
UGA2015 training	UGA2015 testing	0.988	0.995	0.991	0.859	1.000	0.924
U15U18 training	TAMU2015 testing	0.911	0.940	0.925	0.266	0.775	0.396
TAMU2015 training	TAMU2015 testing	0.999	0.999	0.999	0.980	1.000	0.990

### Counting accuracy

**Fig. 5:**
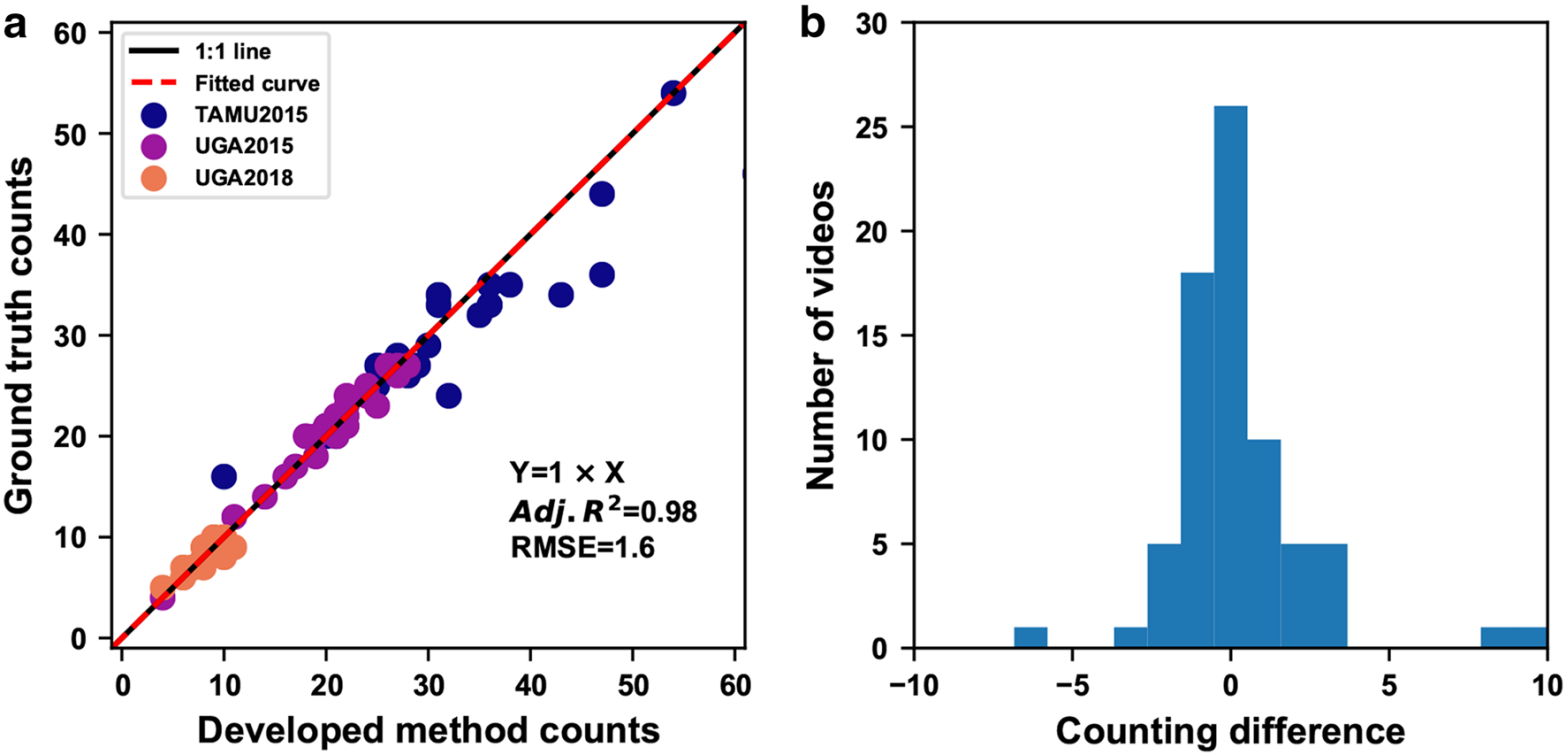
Regression results between seedlings counts calculated by the developed approach and human field assessment. **a** Results obtained using the $model_{SAll}$ detection model for all testing videos (n = 75, lifetime threshold of 7 was used for the TAMU2015 and UGA2018 testing videos and 15 for the UGA2015 testing videos). **b** Counting differences between the proposed method and field assessment. A total of 53 videos (70%) had the counting differences less than 1 seedling, and 68 videos (90%) had the counting differences less than 5 seedlings

Overall, seedling counts that were calculated using the developed approach with the $model_{SAll}$ model were highly correlated ($R^2$ = 0.98) with those by human field assessment (Fig. 5a). The slope of the regression equation was one, suggesting that seedling counts calculated by the developed approach can be used directly. For the 75 testing videos, 53 (70%) videos had an absolute counting error less than or equal to 1 seeding, and 15 (20%) videos with an absolute counting error greater than 1 had a relative counting error of less than 10% (Fig. 5b). Thus, a total of 90% of the testing videos showed acceptable counting accuracies, whereas the rest 10% of the testing videos showed large counting errors (larger than 15%). Nonetheless, the mean relative error for all the 75 videos was 7%, indicating the efficacy of the developed counting approach.

Detection performance showed strong influences on the counting accuracy of the developed approach (Table 3). When detection models (e.g., $model_{T15U15}$ and $model_{U15U18}$) were well generalized to seedling detection, the developed approach with such detection models provided similar counting accuracies for videos collected in separate data collection sessions (see results for TAMU2015 and UGA2018 testing videos in Table 3). It was noteworthy that $R^{2}$ values decreased primarily due to the narrow range of seedling quantities. As a result, subtle counting errors could have considerable influences on the $R^{2}$ values. When detection models (e.g., $model_{T15U18}$ ) had poor generalizability to seedling detection, the counting performance of the developed approach with such models degraded dramatically (see results for UGA2015 testing videos in Table 3).

**Table 3:** Regression results between seedling counts obtained by the proposed method and human field assessment

Detection model	Testing videos	Regression equation	Video quantity	$R^{2}$	RMSE	MAE	MRE (%)
$model_{SAll}$	All	Y = X	75	0.98	1.6	1.5	7
$model_{SAll}$	TAMU2015	Y = 0.96 X	25	0.85	3.3	3.4	11
$model_{SAll}$	UGA2018	Y = X	25	0.99	0.2	0.5	6
$model_{SAll}$	UGA2015	Y = 1.01 X	25	0.96	1.1	0.8	4
$model_{U15U18}$	TAMU2015	Y = 0.95X	25	0.80	3.9	3.4	11
$model_{U15T15}$	UGA2018	Y = 1.02X	25	0.68	0.8	0.6	7
$model_{T15U18}$	UGA2015	Y = 0.32X + 18.65	25	0.33	4.3	12.1	57

$R^{2}$ refers to adjusted $R^{2}$

*RMSE* root mean squared error, *MAE* mean absolute error, *MRE* mean relative error

**Fig. 6:**
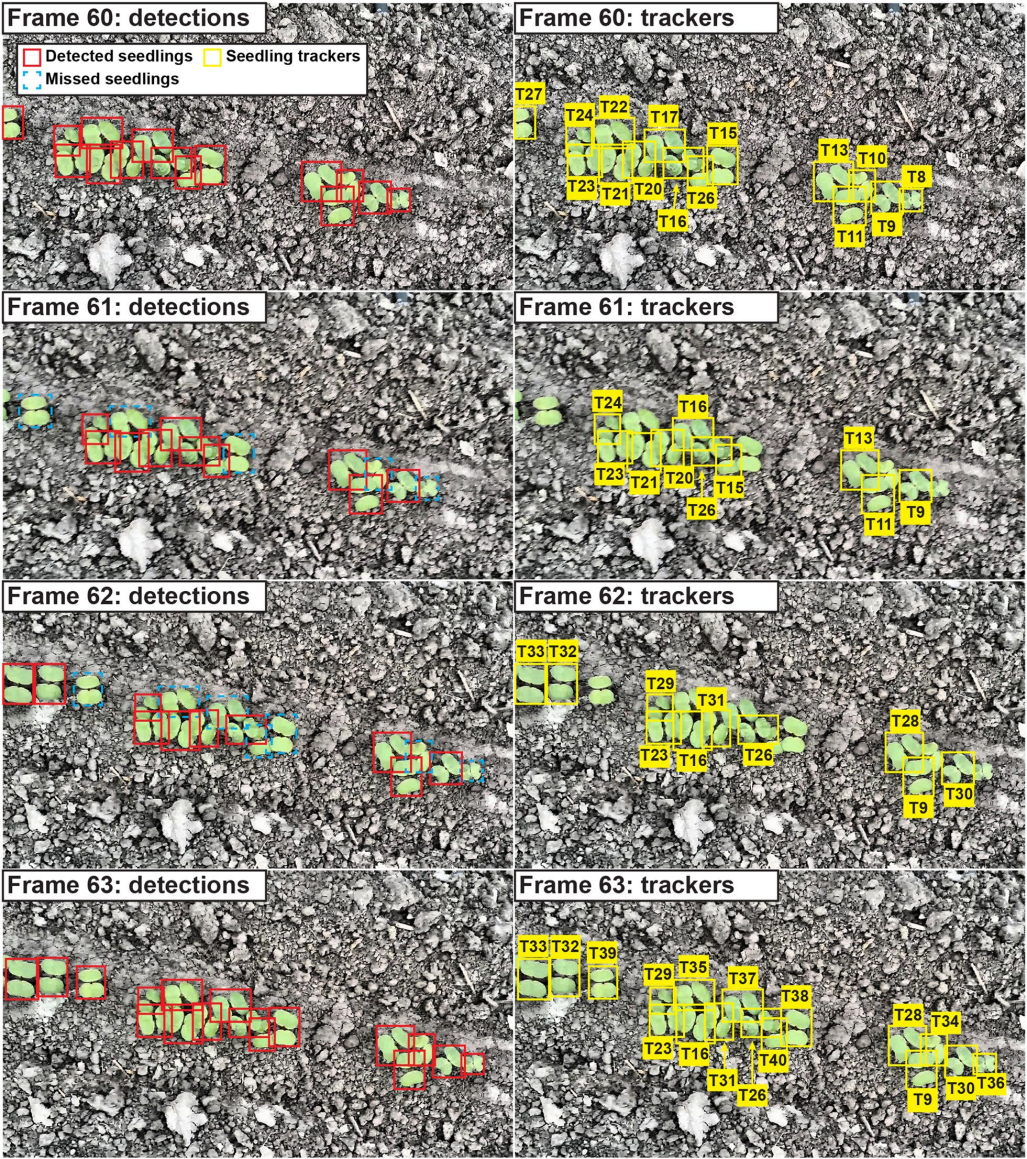
An example of seedling tracking errors due to inaccurate detection results. The detection model was $model_{SAll}$ and the testing video was from TAMU2015 dataset. Detection results were inaccurate in Frame 61 and Frame 62, causing incorrect termination of existing trackers and creation of excessive trackers for some seedlings. These tracking errors eventually led to inaccurate counting results

The reduction of counting accuracy was primarily due to tracking errors caused by inaccurate seedling detection (Fig. 6). In one of the TAMU2015 testing videos, the detection results were accurate in Frame 60 and Frame 63 but not accurate in Frame 61 and Frame 62. Consequently, some trackers lost continuity in tracking between Frame 60 and the following video frames. When all seedlings were correctly detected in Frame 63 again, although some of the detected seedlings were the same in Frame 60 and Frame 63, they were assigned to new trackers due to the tracking discontinuity. As a consequence, the number of seedling trackers would be higher than the actual number of seedlings from Frame 60 to Frame 63, which ultimately led to inaccurate counts of seedlings in that video. Depending on the lifetime of new trackers (the number of frames new trackers could last), the seedling count in a video could be higher or lower than the actual value. This would be a major error source for the developed approach.

## Discussion

### Learned lessons for training seedling detection models

Seedling detection results showed that the selected feature extractor (Inception ResNet v2) should be powerful enough to extract features for differentiation of classes with similar appearance (e.g., cotton seedlings and dicotyledonous weeds). Faster RCNN model, however, did not accurately detect small-sized weeds. In fact, detection of small-sized objects is a general challenging issue for region based neural networks, especially for datasets with a mixture of objects of other sizes [20]. Based on the results, a spatial resolution of 150 $\times$ 150 pixels or higher (in an image of 1920 $\times$ 1080 pixels) would be generally adequate to avoid potential issues due to small-sized objects. Although the used CNN meta-model has the potential for detecting cotton plants in a later stage, overlaps between true leaves would cause significant occlusions between plants, raising a great challenge for object detection. Thus, it suggests that seedling detection needs to be conducted in the cotyledon stage (usually 7–14 days after planting) for the best performance.

Ablation experiment results suggest that model detection performance would be maximized by using optimal training sample size and pre-training/training datasets. Generally, a large number of training samples would benefit the training of Faster RCNN models in terms of localization and classification. The bounding box regressor of Faster RCNN models could be better trained with more training images, providing more accurate localization results (F1 score at $\text {IOU}_{\text {all}}$). Additionally, more training images could allow the learning of adequate feature representations to differentiate classes with a similar appearance, providing dramatic classification performance improvements (e.g., weed detection in UGA2015 dataset).

Transfer learning through weight initialization requires a careful consideration of a dataset used for pre-training. Mostly, datasets collected for the same domain problem are likely similar to each other, so weights pretrained from one dataset could be more beneficial for the model training process for another dataset. For instance, compared with weights pretrained on a common dataset, weights (especially for the bounding box regressor) pretrained on a seedling dataset would be closer to the optimal values for another seedling dataset, resulting in better localization of seedlings. If two datasets for the same domain problem are divergent, transfer learning efficiency is likely to be declined. For instance, the TAMU2015 contained monocotyledonous weeds, whereas the U15U18 contained dicotyledonous weeds. Due to a higher object diversity, weights (features) learned on the U15U18 dataset were insufficient to represent weed objects in the TAMU2015 dataset, resulting in a performance reduction. In such cases, a common dataset (e.g., the COCO dataset) can be used for model pre-training to increase the diversity of learned features and thus the capability of object representation. Training datasets have also demonstrated a considerable effect on the model generalizability. If a training dataset covers all possible object status (e.g., appearance variation and object occlusion), models trained on that dataset usually can be well generalized to new datasets for the same task. Otherwise, model generalizability would be limited. For instance, the T15U15 dataset was diverse to cover possible object status in the UGA2018 dataset, so $model_{T15U15}$ achieved a high accuracy of seedling and weed detection in UGA2018 testing images. Both $model_{T15U18}$ and $model_{U15U18}$, however, showed a poor performance of weed detection for the UGA2015 and TAMU2015 datasets, respectively. This is because their training datasets lacked objects with specific status: the T15U18 dataset contained no dicotyledonous weed that existed in the UGA2015 dataset and the U15U18 dataset contained no monocotyledonous weed that existed in the TAMU2015 dataset.

Training and deployment of deep learning models need the use of high performance computing (HPC) resources. It is crucial to introduce potential ways to access HPC, so researchers and growers (especially small research groups and farms) could benefit from deep learning techniques such as the one presented in this study. Currently, there are three major ways to access HPC resources. First, commercial HPC services are available by transnational companies such as Amazon, Google, and Microsoft. An Amazon GPU node (e.g., p2.xlarge) costs approximately $15 for training/tuning the presented model, which is an affordable solution for small research groups/growers. Second, some universities and research institutions also host HPC clusters that can be used by researchers with a reasonable price. Third, consumer-grade GPUs are typically $200 to $500, which are inexpensive as a long-term investment. In addition, if processing speed is not a required factor (e.g., realtime decision-making), model deployment can use a reduced computing power (e.g., a regular computer), which dramatically lowers down the hardware cost.

### Seedling counting

The developed approach is more efficient and effective than traditional seedling counting methods. High throughput plant phenotyping systems enable a fast and efficient data collection in the field. Based on the presented study, a 10.67-m plot can be imaged in approximately 20 s, and thus a typical experiment involving several hundred of plot can be scanned in 1 or 2 h with only one human operator, which dramatically reduces the labor and time required by traditional methods. On average, the developed approach takes about 3.5 min to process a video of a 10.67-m plot at 30 FPS (equivalent to 0.4 s/frame) using one consumer-grade GPU card (NVIDIA GTX 1080 Ti). Although the processing speed is moderate, it can be further improved by using advanced computing resources (e.g., HPC clusters), optimized computing solutions, and simplified detection models [10].

The developed approach provides a similar accuracy (93%) of seedling counting to other CNN-based approaches for fruit counting [13–15], showing the efficacy of using CNN-based approaches for seedling counting. Compared with the approach based on conventional image processing for seedling counting [7], the developed approach shows two advantages. First, the counting accuracy was improved from 88 to 92%. Second, and more importantly, the developed approach shows great potential to be generic for cotton seedling detection. Experiments showed trained seedling detection models, and the counting approach could be well generalized to unseen datasets, so they can potentially be used in similar applications by the cotton industry and research communities with little or no modification. To the best of our knowledge, the $Seedling_{All}$ dataset is the largest annotated dataset of cotton seedlings, and publicizing such a dataset would benefit both research communities and the cotton industry.

### Limiting factors

While showing certain advantages, the developed approach has two major limiting factors. First, detection models considerably influence the counting accuracy of the developed approach. It is not a trivial task to train an accurate and robust detection model in many applications. Three important factors have been examined in the present study, including the training sample size, transfer learning using different pretrained models, and model generalizability. Experimental results showed that all three factors were somehow data dependent. On one hand, if agronomic practices (e.g., application of pre-emergent herbicides) are implemented in a future project, seedling detection would be fairly simple due to few object categories in videos/images. Thus, seedling detection can be solved using either the $model_{SAll}$ directly, or using a new detection model that is initialized with the $model_{SAll}$ and trained on a small number of annotated images (100 to 300 images based on the present study) from the newly collected dataset. In particular, for a specific experimental site or farm, it is highly recommended to be consistent in data collection conditions (e.g., cameras for image acquisition, camera configuration, and illumination conditions) to enhance the similarity between datasets collected over time and thus counting accuracies for long-term uses. If data in a future project are more complex (e.g., more frequent occlusions, more extreme illumination conditions, and more types of vegetation) than any of the datasets in this study, it is necessary to label a fairly large amount of data to ensure the possibility of achieving the best detection performance. If so, the value of trained models and annotated data in the present study may be relatively reduced in future studies.

Secondly, a conventional Kalman-filter-based tracking algorithm is not adequate to solve issues caused by inaccurate detection of seedlings. When a cotton seedling cannot be correctly detected in consecutive video frames, the current tracking algorithm is likely to terminate the seedling tracker in the frame where that seedling is mis-detected, and assign a new tracker in the next frame where that seedling is re-detected. Thus, that seedling could be counted repeatedly in a video, resulting in counting errors. This occurs primarily because the current strategy of detection/tracker assignment is based on the IOU metric. No detection means no intersection with any existing trackers. There are two ways to solve this issue. First, a new strategy can be used for tracker termination. If no detection can be assigned to a tracker, that tracker can be kept for extended frames (e.g., 3 video frames), which can address the tracking discontinuity due to inaccurate detection results to some extent. In the extended frames, the dynamic model for that tracker is updated using the information obtained in the last frame where that tracker has an associated detection, reducing the model accuracy. Thus, this strategy requires an additional checking procedure that ensures the correctness of detection and tracker association. Feature-based approaches are preferred to maximize the checking accuracy. Second, tracking information can be used to improve detection accuracy. The developed approach solely relies on the detection procedure to provide “ground truth measurements” for trackers. Thus, if the detection procedure is not accurate, the tracking procedure cannot be accurate. To address this issue, the tracking information needs to be used for the detection as well. For instance, the RPN of Faster RCNN could provide inaccurate region proposals (e.g., no region proposal around an existing tracker), leading to misdetection of seedlings and therefore inaccurate tracking. In fact, the tracking procedure predicts bounding boxes of all existing trackers in the next frame. These predicted bounding boxes can be used as region proposals for detection models (e.g., a Faster RCNN model) or be evaluated by a separately trained CNN (e.g., a ResNet model) that determines the presence of plants (classified as either background or plants). With these efforts, it is expected to reduce the possibility of missing an existing tracker in the next frame and ultimately improve the tracking and counting accuracy. This detection-tracking continuum may violate some assumptions of the Kalman filter in a strict consideration. For instance, Kalman filter assumes that sensor measurement is independent of the dynamic model. To solve these potential issues, it is necessary to use other filtering approaches such as the particle filter. These solutions need to be further explored in future studies.

## Conclusions

The developed approach based on deep CNNs can accurately count germinated cotton seedling in the field. Experimental results showed that the approach generalized well to unseen datasets, indicating the great potential of applying the approach for other plant or plant organ detection and tracking. Trained detection models and the annotated images can be reused by the research communities and the cotton industry. Future studies will be focused on the improvement of computation efficiency for real time online processing.

## Methods

### Data collection and preparation

**Fig. 7:**
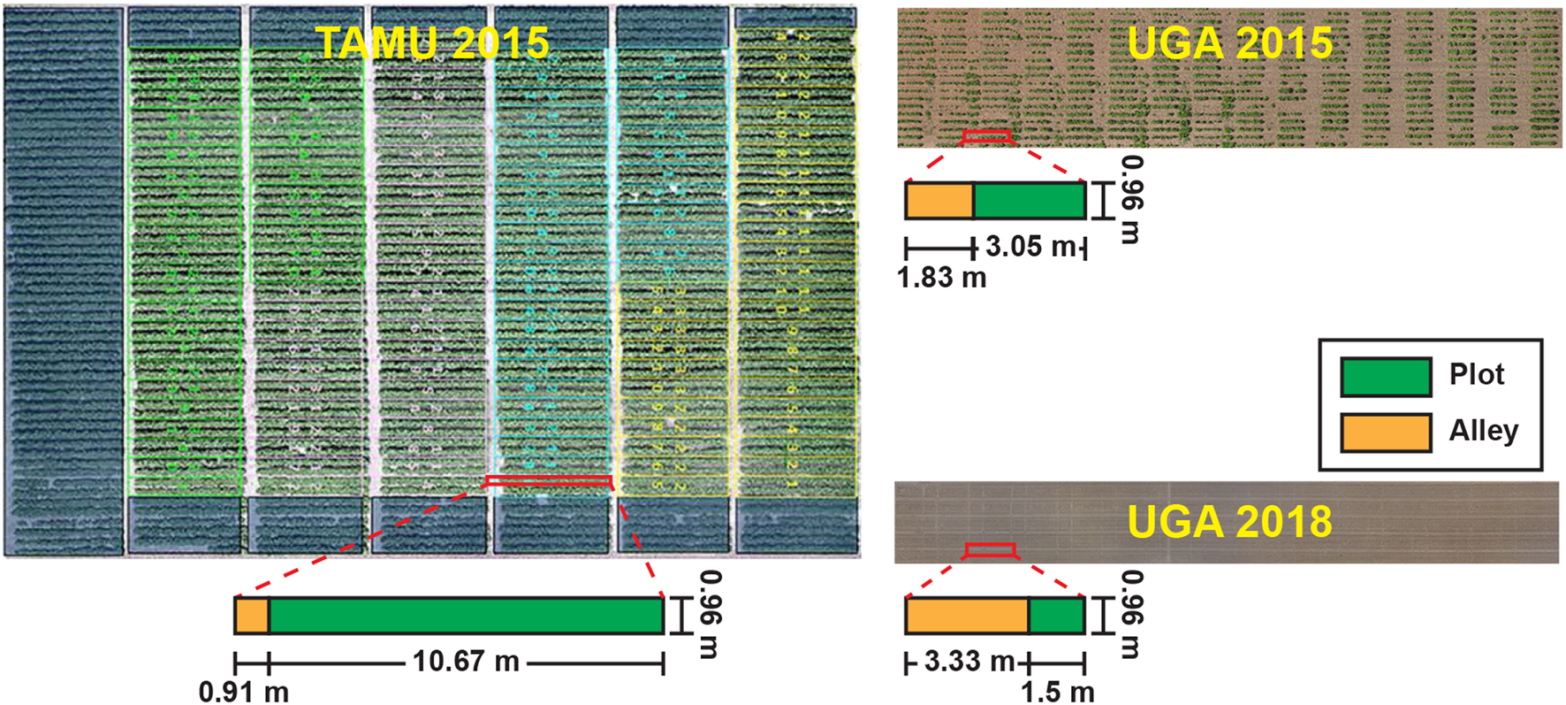
Plot arrangements of different experiment fields used in the presented study. Videos were collected using different cameras in these fields to provide datasets for successive processing

Videos were collected in the cotton germination stage at different locations over multiple years (Table 4). Video collection could be done by a wide range of imaging systems including handheld cameras, moving cart-based systems, and tractor-based systems, which reflects the flexibility of data acquisition for the methodology developed in this study. Different plot arrangements were used in the experiment fields (Fig. 7). Generally, the camera was placed/held at approximately 0.5 m above the ground, minimizing seedling size differences among videos. The collected videos were split into detection and counting sets. Videos in the detection set were used to extract video frames at a frequency of 6 frames per second (FPS), forming three image datasets that were used for plant seedling detection. For convenience, the three image datasets will be referred to as TAMU2015, UGA2015, and UGA2018 hereafter. Videos in the counting set were segregated into 75 video clips (25 clips per dataset) for evaluating the developed counting algorithm. Each video clip represented an approximately 3-m long segment in the videos collected in the state of Texas or a single plot in the videos collected in the state of Georgia.

**Table 4:** Data collection summary

Data collection	Texas A&M University	University of Georgia	University of Georgia
Location	Corpus Christi, TX, USA	Watkinsville, GA, USA	Watkinsville, GA, USA
Plot length	10.67 m	3.05 m	1.5 m
Cultivars	35 commercial cultivars widely grown in Texas	Genotypes in UGA breeding programs	4 commercial cultivars widely grown in Georgia
Seed spacing	0.08 m/seed (average)	0.1 m/seed (average)	0.15 m/seed
Date	12 April 2015 11 days after planting, DAP	15 June 2015 11 DAP	13 June 2018 7 DAP
Weather	Sunny	Cloudy	Sunny
Camera	Samsung Galaxy Note3	Panasonic DMC-G6	Fujifilm X-A10
Video configuration	1920 $\times$ 1080 @ 30 FPS	1920 $\times$ 1080 @ 60 FPS	1920 $\times$ 1080 @ 30 FPS
ISO/HDR	Auto/auto	160/Off	Auto/not support
Average moving speed	0.6 m/s	0.75 m/s	0.6 m/s
Number of collected videos (detection/counting)	3 (2/1)	6 (4/2)	4 (2/2)
Number of plots per video	7	16	19

**Fig. 8:**
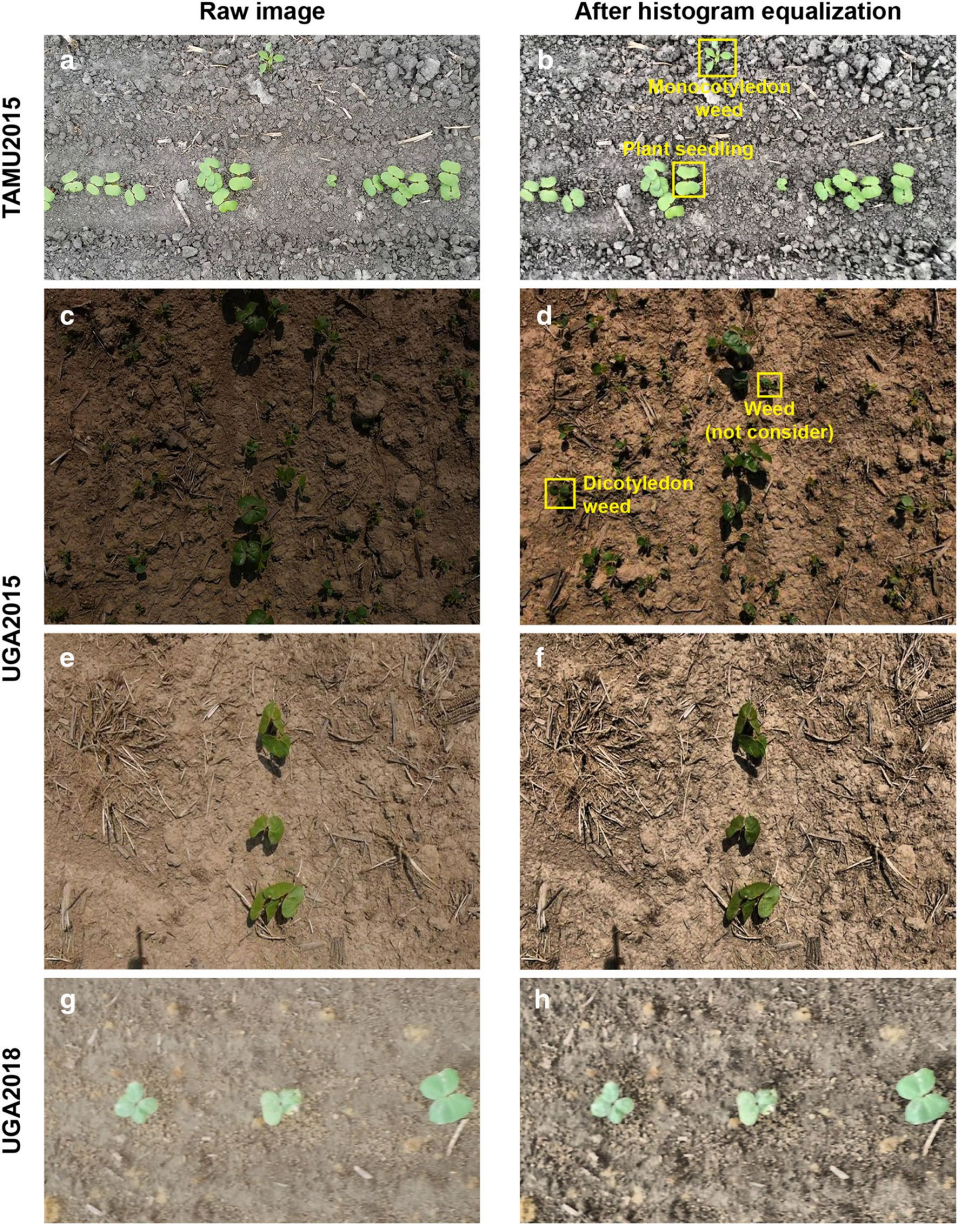
Example images in the TAMU2015, UGA2015, and UGA2018 datasets. For seedling detection, the TAMU2015 dataset shows challenges of high object occlusion and existence of monocotyledonous weed and the UGA2015 dataset shows extreme illumination condition and existence of dicotyledonous weed. On the contrary, the UGA2018 dataset demonstrates a relatively simple and ideal situation for seedling detection

Three image datasets were preprocessed to reduce the image variability (Fig. 8). A contrast limited adaptive histogram equalization (CLAHE) algorithm was applied to equalize the value channel of images in the HSV color space, which enhanced the image contrast and reduced the image variation due to ambient illumination changes. The preprocessed images were manually annotated with bounding boxes for objects of two classes: plant seedlings and weeds. Monocotyledon weed was the only weed type observed and labeled in the TAMU2015 dataset, whereas dicotyledons were the primary weed type in the UGA2015 dataset. Very small-sized weed objects (less than 30 $\times$ 30 pixels) were not labeled. As pre-emergent herbicides were applied, there was no weed identified in the UGA2018 dataset. After manual annotation, the three datasets were partitioned into training, validation, and testing sets with a ratio of 80%/10%/10% (Table 5). Subsequently, four comprehensive datasets were generated by combining the three datasets (Table 6). As the T15U15, T15U18, and U15U18 datasets were only used for model training and validation, the validation and testing sets of the original datasets (e.g., TAMU2015, UGA2015, and UGA2018) were merged into a single validation set. The $Seedling_{All}$ dataset was generated by combining all three datasets with the original partitioning.

**Table 5:** Summary of data annotation and partitioning for the TAMU2015, UGA2015, and UGA2018 datasets

Dataset	TAMU2015	UGA2015	UGA2018
Total number of images	2204	1895	1511
Number of training images	1801	1603	1253
Number of validation images	202	146	129
Number of testing images	201	146	129
Total number of annotations (plant seedling/weed)	21915 (21133/782)	7802 (6849/953)	5880 (5880/0)
Number of training annotations (plant seedling/weed)	17939 (17290/649)	6524 (5743/781)	4862 (4862/0)
Number of validation annotations (plant seedling/weed)	1964 (1911/53)	643 (553/90)	540 (540/0)
Number of testing annotations (plant seedling/weed)	2012 (1932/80)	635 (553/82)	478 (478/0)
Number of videos for counting test	25	25	25

**Table 6:** Summary of data annotation and partitioning for the combined datasets

Dataset	T15U15	T15U18	U15U18	$Seedling_{All}$
Total number of images	4099	3715	3406	5610
Number of training images	3404	3054	2856	4657
Number of validation images	695	661	550	477
Number of testing images	N/A	N/A	N/A	476
Total number of annotations (plant seedling/weed)	29739 (27997/1742)	27809 (27025/794)	13696 (12736/960)	35597 (33862/1735)
Number of training annotations (plant seedling/weed)	24466 (23035/1431)	22805 (22154/661)	11388 (10606/782)	29325 (27895/1430)
Number of validation annotations (plant seedling/weed)	5273 (4962/311)	5004 (4871/133)	2308 (2130/178)	3147 (3004/143)
Number of testing annotations (plant seedling/weed)	N/A	N/A	N/A	3125 (2963/162)

### Faster RCNN for seedling detection

#### Model architecture, training, and evaluation

The Faster RCNN meta architecture was used due to its success in many object detection applications [10]. The architecture contains a feature extractor, a region proposal network (RPN), and a classification and regressor module. The feature extractor is usually a deep CNN network, which extracts informative feature representations from the raw input images in a hierarchical fashion. The RPN uses the extracted features to generate potential regions of interest (ROIs), and the classification and regressor module uses the features in each ROI to identify the ROI class and refine the coordinates of ROI bounding box. In this study, the Inception ResNet v2 network [21] was used as the feature extractor due to its great potential of differentiating classes with similar appearances and shapes (e.g., dicotyledonous weed and cotton seedlings).

Transfer learning was used to improve the training efficiency and effectiveness. In the present study, the Faster RCNN model was initialized by weights pretrained on the common objects in contexts (COCO) dataset, and fine-tuned on the $Seedling_{All}$ training set. Mini-batch stochastic gradient descent (SGD) and the Adam optimizer were used for model training. While training the model, data augmentation was performed to increase the diversity of training images, including horizontal and vertical image flip and random changes of image saturation, brightness, and contrast. The Faster RCNN model and training programs were implemented using Tensorflow. Training processes were performed on two computing nodes hosted by the Georgia Advanced Computing Resource Center (GACRC), with each being configured with 14 CPU cores (2.8 GHz per core), 120 GB CPU memory, and a GPU card (Tesla V100 16 GB, NVIDIA Corporation, Santa Clara, CA, USA) under the operating system of CentOS 7.5. Based on preliminary experiments, the model was trained for a total of 50,000 iterations (equivalent to 22 epochs) using an initial learning rate of 0.0001, a dropout rate of 0.5 for the RPN and classification and regressor modules, and weight decay of 0.001. Model checkpoints were saved after every 5000 training iterations, and a total of 10 checkpoints were evaluated on the validation set to select the best model for testing and seedling counting. For the sake of brevity, the training procedure and configuration was the base training configuration.

Mean average precision (mAP), mean average recall of the top 100 detections (mAR100), and an F1 score were calculated at $IOU_{0.5}$ and $IOU_{all}$ (from $IOU_{0.5}$ to $IOU_{0.95}$ with an interval of 0.05) and used as metrics to evaluate the overall performance of detection models. The use of metrics at $IOU_{all}$ was to more strictly evaluate the localization accuracy of detection models. In addition, average precision (AP), average recall of the top 100 detections (AR100), and an F1 score at $IOU_{0.5}$ were calculated for the seedling and weed classes, so per-category performance of detection could be analyzed. These evaluation metrics were used for ablation experiments as well.

#### Ablation experiments

Many practices could be used to optimize the training of CNNs and CNN meta-models. In this study, three aspects were investigated to provide an optimal configuration to train Faster RCNN models for seedling detection, including training sample size, transfer learning with different pretrained models, and model generalizability.

*Training sample size* While model performance benefits from a large amount of training samples, it is usually laborious to obtain a large training set for domain applications such as seedling detection. Therefore, it is important to investigate improvements of model performance due to increased training sample sizes. For each of the TAMU2015, UGA2015, and UGA2018 datasets, a total of 7 Faster RCNN models were trained using different training sample sizes, including 100, 200, 300, 500, 700, and 1000 randomly selected training images and all training images. Model training was conducted using the base training configuration, with the reduction of training iterations from 50,000 to 35,000 (based on preliminary experiments). For each training sample size, the best model checkpoint was selected based on validation performance and was used to obtain testing performance.

*Transfer learning using different pretrained models* In the present study, transfer learning was implemented through model initialization using pretrained weights. Thus, pretrained models may have considerable impact on the transfer learning efficiency. Model initialization using weights pretrained on large common datasets (e.g., ImageNet and COCO) would improve the model training for domain applications, especially for those with a small number of training images. However, improvements could be degraded when domain data are extremely different from the common datasets. Two model initialization methods were used and compared to examine the transfer learning efficiency using different pretrained models: (1) model initialization using weights pretrained on the COCO dataset and (2) model initialization using weights pretrained on a domain dataset that is different from a target dataset. For example, if the UGA2018 dataset was a target dataset, Faster RCNN models would be initialized using weights pretrained on the COCO and T15U15 datasets, respectively. Subsequently, the two initialized models were trained on a subset of 100 images that were randomly selected from the UGA2018 training set. The base training configuration was used for model training and validation, with the reduction of training iterations from 50,000 to 5000 (based on preliminary experiments). Performance on the UGA2018 testing set was obtained using the best checkpoint for each of the two models. This process was repeated 10 times, so a total of 10 testing results were calculated for each initialization method for a given target dataset, enabling statistical comparisons between the two methods. The TAMU2015 and UGA2015 were used as target datasets as well.

*Model generalizability* The model generalizability was also a key factor for training deep neural networks due to the high cost of labeling a large amount of images for domain applications. To evaluate model generalizability, detection performance on a target dataset was compared between models trained using datasets acquired in the same and different data collection sessions. For instance, if the TAMU2015 testing set was a target dataset, detection performance of a Faster RCNN model trained on the TAMU2015 training set would be compared with that trained on the U15U18 dataset. The base training configuration was used for model training and validation.

### DeepSeedling framework for seedling counting

**Fig. 9:**
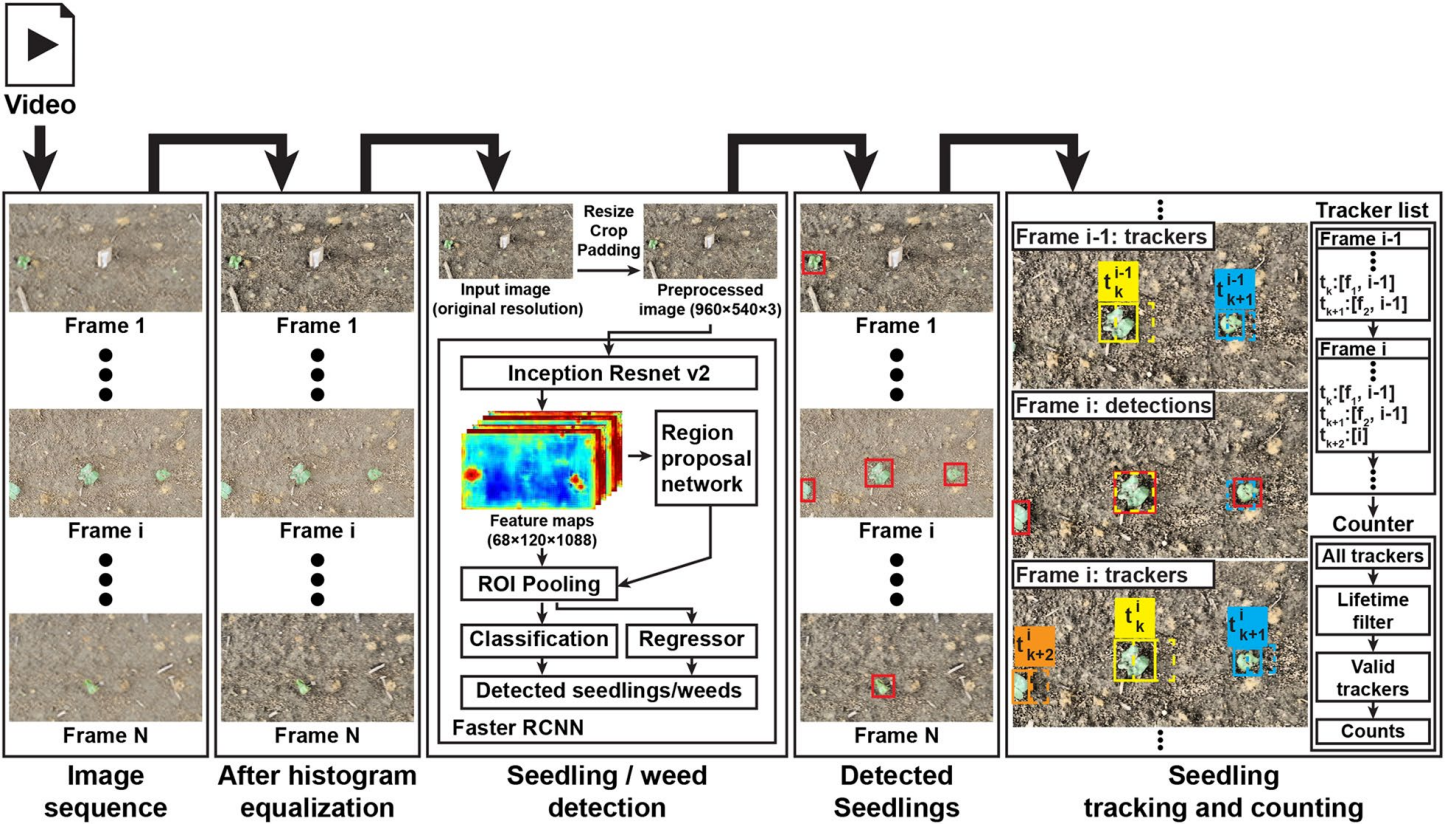
Flowchart of the deep convolutional network based approach for cotton seedling detection and counting. After preprocessing, video frames are fed into a trained Faster RCNN model for seedling detection. Detected seedlings are associated using a Kalman filter-based video tracking algorithm, so the same plant object will be tracked through video frames and not counted repeatedly

With an optimal configuration, a Faster RCNN model was obtained for seedling detection in static images. DeepSeedling framework was developed to integrate the trained Faster RCNN model for cotton seedling detection and counting in videos (Fig. 9). For a given video of cotton seedlings, video frames were extracted at the video frame rate and enhanced using the CLAHE algorithm. The enhanced images were fed into a Faster RCNN model to detect cotton seedlings. The detected seedlings were tracked in all video frames to count the number of seedlings in the given video. The key concept of the framework was to use computer vision techniques to track seedlings detected in video frames, avoiding repeated counting of the same seedling object.

#### Seedling counting by tracking

The total number of detected seedlings from all frames of a video was considerably larger than the actual number of seedlings in that video, because one cotton seedling could be repeatedly detected in consecutive video frames, resulting in recurrent counts. To address this issue, detected seedlings were tracked in a video, so each seedling would be assigned with a single tracker and thus counted only once.

In the present study, seedling tracking was essentially to associate multiple detections (bounding boxes) of the same seedling over consecutive frames in a video. The state of a seedling tracker ($\pmb {t}$) included the detection and moving speed of a seedling in a video, and was formulated using Eq. 1.

1 $$\pmb {t} = [u, v, s, r, {\dot{u}}, {\dot{v}}, {\dot{s}}]^T$$

where *u*, *v*, *s*, *r* were the horizontal and vertical center (in pixels), area (in pixels), and aspect ratio of a bounding box. ${\dot{u}}, {\dot{v}}, {\dot{s}}$ were the corresponding first-order derivatives with respect to time (in the unit of video frames).

The Kalman filter [22] was used to track detected seedlings in consecutive video frames (object tracking and counting in Fig. 9). The seedling tracking was treated as a discrete-time filtering problem, and it was solved by two steps [23]. The first step was prediction process (also known as time update) in which states of seedling trackers in the current frame were used to predict their states in the next frame using the dynamic model of Kalman filter. The second step was update process (also known as measurement update) in which observations (seedling detections) in the next frame were associated with the seedling trackers to update the tracker states and the dynamic model of Kalman filter. The two steps were performed alternatively over frames to track seedlings in a video.

In the first frame ($i = 1$), seedling trackers ($T_{1}$) were initialized using the seedling detections identified by a Faster RCNN model, with a value of zero for ${\dot{u}}, {\dot{v}}, {\dot{s}}$. Starting from the second frame ($i \ge 2$), tracker states ($\pmb {t}$) and the state covariance matrix ($\pmb {P}$) in the *i*th frame were estimated using the information of seedling trackers in the *i* − 1th frame by the prediction process (Eqs. 2 and 3).

2 $$\begin{aligned} \pmb {{\hat{t}}^{i|i-1}_{k}} & = \pmb {F}\pmb {{\hat{t}}^{i-1|i-1}_{k}},\; \pmb {F} = \begin{bmatrix} 1&0&0&0&1&0&0\\ 0&1&0&0&0&1&0\\ 0&0&1&0&0&0&1\\ 0&0&0&1&0&0&0\\ 0&0&0&0&1&0&0\\ 0&0&0&0&0&1&0\\ 0&0&0&0&0&0&1 \end{bmatrix} \end{aligned}$$

3 $$\begin{aligned} \pmb {P_{i|i-1}} & = \pmb {F}\pmb {P_{i-1|i-1}}\pmb {F^{T}} + \pmb {Q}, \; \pmb {Q} = \begin{bmatrix} 1&0&0&0&0&0&0\\ 0&1&0&0&0&0&0\\ 0&0&1&0&0&0&0\\ 0&0&0&1&0&0&0\\ 0&0&0&0&10^{-2}&0&0\\ 0&0&0&0&0&10^{-2}&0\\ 0&0&0&0&0&0&10^{-4} \end{bmatrix} \end{aligned}$$

where $\pmb {{\hat{t}}^{i|i-1}_{k}}$ was the a priori estimated state of the *k*th seedling tracker in the *i*th frame, $\pmb {{\hat{t}}^{i-1|i-1}_{k}}$ was the a posteriori estimated state of the *k*th seedling tracker in the *i* − 1th frame, and $\pmb {F}$ was the matrix of state transition. $\pmb {P_{i|i-1}}$ was the a priori state covariance matrix for the *i*th frame, and $\pmb {P_{i-1|i-1}}$ was the a posteriori state covariance matrix for the *i* − 1th frame. $\pmb {Q}$ was the process noise covariance matrix and determined arbitrarily in this study [23].

In the *i*th frame, the Kalman filter was updated by the update process using seedling trackers in the *i* − 1th frame ($T_{i-1}$) and seedling detections in the current frame ($D_{i}$). As detections were the ground truth measurements for existing trackers, it was necessary to associate detections and trackers for updating the Kalman filter. The cost of assigning a new detection ($\pmb {d_{j}} \in D_{i}$) to an existing tracker ($\pmb {t_{k}} \in T_{i-1}$) was the negative IOU value between the detection ($\pmb {d_{j}}$) and the tracker’s predicted detection ($\pmb {{\hat{t}}^{i|i-1}_{k}}$). The assignment task was optimally solved using the Hungarian algorithm [24] that minimized the assignment cost under a constraint of the minimum IOU value of 0.1 (Eq. 4).

4 $$\begin{aligned} \begin{array}{lll} \min & {\sum\limits _{j=1}^{N(D_{i})}} {\sum\limits_{k=1}^{N(T_{i-1})}} c(\pmb {d_{j}}, \pmb {{\hat{t}}^{i|i-1}_{k}})a_{j, k}, \quad a_{j, k} = \left\{ \begin{array}{ll} 1, & \quad \pmb {d_{j}} \; assigned \; to \; \pmb {{\hat{t}}^{i|i-1}_{k}}\\ 0, & \quad otherwise \end{array}\right. \\ subject \; to & \forall j, \quad a_{j, k_{1}} = a_{j, k_{2}} \Rightarrow k_{1} = k_{2}, \quad k_{1}, k_{2} = 1, \dots , N(T_{i-1}) \\ & \forall k, \quad a_{j_{1}, k} = a_{j_{2}, k} \Rightarrow j_{1} = j_{2}, \quad j_{1}, j_{2} = 1, \dots , N(D_{i}) \\ & \forall j \; and \; k, \quad |c(\pmb {d_{j}}, \pmb {{\hat{t}}^{i|i-1}_{k}})| > 0.1 \end{array} \end{aligned}$$

where $D_{i}$ was the set of seedling detections in the *i*th frame and $T_{i-1}$ was the set of trackers in the *i* − 1th frame. $N(\cdot )$ was the function counting the number of elements in a set. $\pmb {d_{j}}$ was the state of the *j*th seedling detection in $D_{i}$ and $\pmb {{\hat{t}}^{i|i-1}_{k}}$ was the a priori estimated state of the *k*th tracker ($\pmb {t_{k}}$) in $T_{i-1}$. $c(\pmb {d_{j}}, \pmb {{\hat{t}}^{i|i-1}_{k}})$ was the cost for assigning $\pmb {d_{j}}$ to $\pmb {{\hat{t}}^{i|i-1}_{k}}$ (i.e., the negative IOU value between the bounding boxes of $\pmb {d_{j}}$ and $\pmb {{\hat{t}}^{i|i-1}_{k}}$), and $a_{j, k}$ indicated the assignment flag, with one for assigning $\pmb {d_{j}}$ to $\pmb {{\hat{t}}^{i|i-1}_{k}}$. It should be noted that one detection could be only assigned to one tracker or otherwise unassigned.

After the detection-tracker association, the detections ($D_{i}$) and trackers ($T_{i-1}$) were categorized into three groups: trackers associated with new detection, unassigned detections, and unassociated trackers. Trackers associated with new detection were used for the update process that calculated the a posteriori state covariance matrix in the *i*th frame ($\pmb {P_{i|i}}$) using Eqs. 5–7 and their a posteriori states of the trackers using Eqs. 8 and 9.

5 $$\begin{aligned} \pmb {S_{i}} & = \pmb {H}\pmb {P_{i|i-1}}\pmb {H^{T}} + \pmb {R}, \; \pmb {H} = \begin{bmatrix} 1&0&0&0&0&0&0\\ 0&1&0&0&0&0&0\\ 0&0&1&0&0&0&0\\ 0&0&0&1&0&0&0 \end{bmatrix}, \; \pmb {R} = \begin{bmatrix} 1&0&0&0 \\ 0&1&0&0 \\ 0&0&10&0 \\ 0&0&0&10 \end{bmatrix} \end{aligned}$$

6 $$\pmb {K_{i}}= \pmb {P_{i|i-1}}\pmb {H^{T}}\pmb {S_{i}^{-1}}$$

7 $$\pmb {P_{i|i}} = (\pmb {I} - \pmb {K_{i}}\pmb {H}) \pmb {P_{i|i-1}}(\pmb {I} - \pmb {K_{i}}\pmb {H})^{T} + \pmb {K_{i}}\pmb {R}\pmb {K_{i}^T}$$

8 $$\pmb {y^{i}}= \pmb {d^{t}} - \pmb {H}\pmb {{\hat{t}}^{i|i-1}_{associated}}$$

9 $$\pmb {{\hat{t}}^{i|i}_{associated}}=\pmb {{\hat{t}}^{i|i-1}_{associated}} + \pmb {K_{i}}\pmb {y^{i}}$$

where $\pmb {S_{i}}$ was the innovation covariance matrix of the Kalman filter in the *i*th frame. $\pmb {H}$ was the measurement matrix that mapped a tracker state to a measurement (detection) state. $\pmb {R}$ was the measurement error covariance matrix and determined arbitrarily in this study. $\pmb {K_{i}}$ and $\pmb {I}$ were the optimal Kalman gain for the *i*th frame and identity matrix, respectively. $\pmb {y^{i}}$ was the innovation (also known as measurement residual) between the a priori estimated state of a tracker ($\pmb {{\hat{t}}^{i|i-1}_{associated}}$) and the state of that tracker’s associated detection ($\pmb {d^{t}}$) in the *i*th frame, and $\pmb {{\hat{t}}^{i|i}_{associated}}$ was the a posteriori estimated state of that tracker.

Unassigned detections (zeros for ${\dot{u}}, {\dot{v}}, {\dot{s}}$) were initialized as new trackers and added into the existing tracker set ($T_{i-1}$). Unassociated trackers from $T_{i-1}$ were removed, forming the new tracker set for the *i*th frame ($T_{i}$). All frames were processed sequentially using the prediction and update processes of the Kalman filter, which provided a list of trackers with their lifetime (number of video frames in which trackers existed).

In theory, the number of trackers would be the number of seedlings in a video as one tracker exactly corresponded to one seedling. However, in practice, seedling detection models could occasionally provide inaccurate detection results, resulting in potential initialization of noisy trackers. Thus, a lifetime filter was used to select valid trackers (trackers with a lifetime longer than a threshold), and the number of valid trackers was used as the number of seedlings in a video. In the present study, the lifetime threshold was arbitrarily set as a quarter of video frame rate, which was 7 for TAMU2015 and UGA2018 videos and 15 for the UGA2015 testing videos.

#### Counting accuracy evaluation

The developed approach with $model_{SAll}$ was used to count the number of seedlings in the 75 testing videos collected in multiple locations and years. Simple linear regression tests were performed between the video-derived counts and human field assessment, and the fitted slope, adjusted coefficient of determination ($R^2$), and root mean squared error (RMSE) were used as the evaluation metrics. Mean absolute error (MAE) and mean relative error (MRE) were also calculated as additional metrics for counting accuracy evaluation. In addition, the developed approach with $model_{U15U18}$, $model_{U15T15}$, and $model_{T15U18}$ was tested using 25 testing videos of TAMU2015, UGA2018, and UGA2015, respectively. The same metrics were calculated to compare with those calculated using $model_{SAll}$, which provided an evaluation of the counting accuracy of unknown datasets.

## Data Availability

Original images and annotations, source code, and testing videos are archived in a GitHub repository (https://github.com/UGA-BSAIL/deepseedling) along with the instruction to run pretrained models for seedling detection and counting.

## Abbreviations

AP: average precision
AR100: average recall given 100 detections per image
CLAHE: contrast limited adaptive histogram equalization
CNN: convolutional neural network
COCO: common objects in context, which is a large-scale object detection, segmentation, and captioning dataset
F1 score: the harmonic mean of precision and recall for a classification problem
FPS: frames per second
GPS: global positioning system
HPC: high performance computing
IMU: inertial measurement unit
IOU: intersection over union, which is the ratio of intersection area and union area between two bounding boxes
MAE: mean absolute error
mAP: mean average precision
mAR100: mean average recall given 100 detections per image
MRE: mean relative error
RCNN: region-based convolutional neural network
ROI: region of interest
RPN: region proposal network
RTK: real time kinematic
SfM: structure from motion technique
SGD: stochastic gradient descent
